# A Survey of MAC Protocols for Cognitive Radio Body Area Networks

**DOI:** 10.3390/s150409189

**Published:** 2015-04-20

**Authors:** Sabin Bhandari, Sangman Moh

**Affiliations:** Department of Computer Engineering, Chosun University, 309 Pilmun-daero, Dong-gu, Gwangju 501-759, Korea; E-Mail: sabinbhd@gmail.com

**Keywords:** wireless body area network, cognitive radio body area network, media access control, energy efficiency, network lifetime

## Abstract

The advancement in electronics, wireless communications and integrated circuits has enabled the development of small low-power sensors and actuators that can be placed on, in or around the human body. A wireless body area network (WBAN) can be effectively used to deliver the sensory data to a central server, where it can be monitored, stored and analyzed. For more than a decade, cognitive radio (CR) technology has been widely adopted in wireless networks, as it utilizes the available spectra of licensed, as well as unlicensed bands. A cognitive radio body area network (CRBAN) is a CR-enabled WBAN. Unlike other wireless networks, CRBANs have specific requirements, such as being able to automatically sense their environments and to utilize unused, licensed spectra without interfering with licensed users, but existing protocols cannot fulfill them. In particular, the medium access control (MAC) layer plays a key role in cognitive radio functions, such as channel sensing, resource allocation, spectrum mobility and spectrum sharing. To address various application-specific requirements in CRBANs, several MAC protocols have been proposed in the literature. In this paper, we survey MAC protocols for CRBANs. We then compare the different MAC protocols with one another and discuss challenging open issues in the relevant research.

## 1. Introduction

In recent times, advancements in wireless sensor networks have enabled them to support a wide range of applications, including medical and healthcare systems. A wireless body area network (WBAN) is a special-purpose sensor network designed to operate autonomously to connect medical sensors and appliances located inside and outside the human body and is used for long-term health monitoring within a hospital or remotely. A WBAN consists of biomedical sensor nodes used to monitor physiological data, such as temperature, blood pressure, electrocardiogram (ECG), electroencephalography (EEG), heart rate, *etc.* [1]. The key requirements of WBANs are low power consumption, negligible electromagnetic interface with the body, low delay, high reliability and effective communication. With regard to the location of the sensors that collect physiological data, the areas inside and around the patient’s body pose a challenging environment for the design of adaptable, dynamic and flexible protocols in WBANs. For the successful implementation of WBANs in medical, as well as consumer products, a standard protocol is required.

The IEEE 802.15.6 standard [2] defines the physical (PHY) and medium access control (MAC) layers to address both medical/healthcare applications and other non-medical applications with varying requirements. The MAC layer in the standard is used to define short-range wireless communication in and around the human body. The standard aims to support a low-complexity, low-cost, ultra-low power and highly reliable wireless communication for use in close proximity to or inside the human body (but not limited to humans) in order to satisfy an evolutionary set of entertainment and healthcare products and services. It is notable that many parts of the MAC protocol in the IEEE 802.15.6 standard are derived from the IEEE 802.15.4 standard [3], because of the similarities between wireless personal area networks (WPANs) and WBANs.

Because of the spectrum scarcity problem recently encountered in wireless communication domains, cognitive radio (CR) technology has emerged as a key technique in wireless networks, including fifth-generation mobile networks. CR technology is a promising technique for unlicensed users to access underutilized licensed (or white space) spectra. It enables unlicensed users (or secondary users (SUs)) to opportunistically access underutilized licensed spectra whenever the licensed users (or primary users (PUs)) are idle [4]. The MAC layer plays a key role in several cognitive radio functions, such as channel sensing, resource allocation, spectrum mobility and spectrum sharing. Channel sensing is the ability of a cognitive user (an SU) to estimate the channel state information and to analyze the radio environment. Resource allocation is the opportunistic assignment of available channels to cognitive users (SUs) according to quality of service (QoS) requirements. Spectrum mobility allows an SU to hand off its assigned channel when a PU is detected and to access a vacant channel to re-establish communication. Spectrum access can help avoid conflicts between primary and secondary users to avoid harmful interference.

CR technology can be implemented in WBANs to reduce interference caused by conventional wireless medical applications. Electromagnetic interference (EMI) may cause malfunction (e.g., wave distortion, shutdown, restart, *etc.*) in the network, which critically affects the operation and communication of various sensitive devices [5]. In cognitive radio body area networks (CRBANs), the transmission parameters of SUs can be adjusted based on EMI constraints to overcome the interference with PUs. CR technology can also improve the QoS of wireless communication between medical devices by defining the priority levels of each device [6]. Cognitive tasks are performed by different radio modules, most of which are directly controlled by the MAC layer.

In [7], the key design features, standard radio technologies and challenges in WBANs are extensively addressed. Furthermore, the MAC layer challenges, energy consumption, coexistence and issues concerning channel modeling are also analyzed and summarized. An interesting and systematic study of a machine-to-machine (M2M) system for mobile health (mHealth) applications is introduced in [8]. That is, the different PHY and MAC design approaches to develop efficient mHealth applications for WBANs are surveyed and discussed. In addition, the integration challenges between diverse communication technologies and highlighted different design approaches for end-to-end connectivity through some examples of practical implementations are presented. On the other hand, a comprehensive review of the different MAC protocols developed for WBANs is presented in [9]. The review discusses the design requirements of the MAC protocols for WBANs with emphasis on energy minimization and also investigates the existing protocols with a focus on their strength and weakness.

A number of MAC protocols have been proposed for WBANs, but few have been developed for CRBANs. Therefore, the development of a MAC protocol for CRBANs is a promising area of research. In this paper, we survey and compare recent advances and development trends in MAC protocols for CRBANs and then address open research issues and challenges.

The rest of this paper is organized as follows: in Section 2, we briefly discuss important MAC design issues in CRBANs. Section 3 is devoted to a review of existing MAC protocols for CRBANs with regard to significant protocol features. In Section 4, we compare the reviewed MAC protocols in detail. Crucial open research issues are discussed in Section 5, and we offer our conclusions in Section 6.

## 2. MAC Design Issues in CRBANs

Cognitive radio is a new technology that enables the more flexible and efficient use of the radio spectrum. It allows unlicensed users to access the radio spectrum without harmful interference with licensed users. A CR device intelligently adapts its spectrum usage by changing radio frequency according to predefined learning parameters to select the best operating frequency and transmission parameters. The industrial, scientific and medical (ISM) band is shared by various technologies, such as IEEE 802.11 (Wi-Fi), IEEE 802.15.1 (Bluetooth) and IEEE 802.15.4 (ZigBee). These technologies operate and coexist in the same frequency band, which may cause interference between different radio systems.

Issues related to CR can span all layers of the communication protocol stack. The main functions of CR are spectrum sensing, spectrum access and spectrum sharing and are mostly performed by the PHY and MAC layers. There are several differences between the CR-aware MAC protocol and traditional MAC protocols. One is the number of available channels, which varies with both time and spatial dimensions in CR networks, but is fixed for each user in traditional networks. Furthermore, in CR networks, the MAC protocols should handle interference with PUs to protect them [10]. MAC protocols play an important role in exploiting spectrum opportunities, collision avoidance, interference control and avoidance for PUs and coordinating spectrum access to SUs. The main issues that must be addressed by CR-aware MAC in CRBANs are as follows.

### 2.1. Spectrum Access

The most important issue pertaining to the MAC in CRBANs is to prevent SUs from colliding and interfering with PUs. Self-coexistence is difficult to achieve, because of the non-deterministic activities of PUs and other noise. Effective spectrum access can be attained in underlay or overlay approaches. In the underlay approach, the SUs employ a combination of lower power transmission and wider frequency bandwidth to generate a signal that appears as noise to the PUs. The spectrum handovers are not issued, because the transmissions of SUs are perceived to be noise by the PUs. On the other hand, overlay access generally uses higher transmission power and a narrower transmission bandwidth. This approach requires accurate and appropriate sensing and signaling mechanisms to handle PU behavior. Coordinated spectrum access provides better spectrum utilization and less interference than uncoordinated spectrum access, because of the cooperation between radios through the exchange of real-time spectrum information.

### 2.2. Energy Efficiency

There are several attributes to be considered for the design of an effective, reliable and energy-efficient MAC protocol. The main goal is to achieve energy efficiency. In CRBANs, all sensors and actuators are operated by a small battery. For long-term operation, it is necessary to minimize energy dissipation. To achieve this goal, energy-aware communication protocols need to be designed. In general, energy efficiency can be achieved by controlling and minimizing waste. Research [11,12] has shown that the main source of energy wastage is data collisions, overhearing, packet overhead, traffic fluctuation and idle listening.

### 2.3. Cross-Layer Design

Spectrum sensing is carried out by the PHY and MAC layers, but spectrum management (such as spectrum decision making, scheduling and spectrum handover) can be related to all other network layers. Therefore, different layers of the protocol stack should be coordinated. As in other wireless networks, network performance and reliability can be significantly improved in CRBANs by considering the cross-layer design and optimization.

### 2.4. Opportunistic Sensing

SUs sense a channel whenever they have the opportunity and maintain a list of empty channels that is independent of other nodes [13]. In opportunistic sensing, the CR node transmits a data packet without sensing the relevant channel by selecting it from the empty channel list; hence, it experiences a short transmission delay. To effectively sense the entire channel, a node should collaborate with other nodes to adjust the sensing priorities of the channel in question.

### 2.5. Optimized Spectrum Decision

SUs dynamically choose the best available channels and transmission parameters. CR-aware MAC protocols should be able to choose the best spectrum using minimum time and energy. Determining the spectrum is among the main design issues in CRBANs. Several techniques and methods can be applied for the learning and decision making processes, such as evolutionary computation, fuzzy logic and the Markov decision process [14]. Nonetheless, more advanced schemes are needed for optimized spectrum decisions.

## 3. MAC Protocols for CRBANs

In this section, we review existing MAC protocols for CRBANs and highlight them with regard to their key characteristics and features.

### 3.1. CR-Based MAC Protocol for Cognitive Wireless Sensor Body Area Networking

In the CR-based MAC (CR-MAC) protocol for wireless sensor body area networks [15], a separate type of sensor node is used for critical health information and non-critical health information. In this protocol, the network nodes are assumed to have the capability to dynamically adjust their transmission power according to the level of urgency of the traffic. The CR-MAC protocol regulates access by critical and non-critical packets to the transmission medium by transmitting packets with higher and lower transmission powers, respectively. The receiver circuit at the PAN coordination node enables the successful reception of critical packets with higher power, even though other, lower priority packets with a lower power are simultaneously received.

In CR-MAC, three transmission power levels of *P_1_*, *P_2_* and *P_3_* are assigned to non-critical traffic, moderately urgent traffic and critical traffic, respectively, where *P_1_* < *P_2_* < *P_3_*. Critical traffic has the highest priority, and moderately urgent traffic has the second-highest. The successful transmission of a moderately urgent packet using power level P_2_ occurs as long as there are no transmitted packets of higher priority in the traffic. Non-critical traffic is subsequently transmitted using power level P_1_, and the successful transmission of packets requires only a single packet in a time slot.

The CR-MAC protocol was analyzed mathematically for two transmission power levels in [15]. In the analysis, a star topology composed of a number of different sensor nodes and a single network coordinator was used. The different sensor nodes are composed of reduced function devices (RFDs), and the coordinator is a full function device (FFD). The RFD nodes are classified into critical and non-critical sensor nodes according to their assigned physiological variable of measurement, such as EEG, ECG, heartbeat, blood pressure, temperature, *etc.* The RFD nodes periodically report the measured physiological values to the FFD coordinator node, which transfers the data to the server through other networks, such as cellular systems or wireless local area networks (WLANs), where the data are monitored and analyzed.

Critical traffic throughput is first analyzed because critical traffic transmission is independent of non-critical traffic transmission. The output is then applied to the analysis of non-critical traffic throughput, which depends on the transmission of critical traffic. As an essential parameter, the traffic rejection rate for critical and non-critical packets was also analyzed in [15]. The probability of critical traffic rejection depends on the number of allowed critical packet retransmissions. For the transmission of the first packet, the probability of packet transmission failure is $\left(1-p_{c}\right)$, where $p_{c}$ is the probability of a successful critical packet transmission in a particular time slot. The probability of critical packet transmission failure is ${\left(1-\;p_{c}\;\right)}^{r_{c}+1}$, where $r_{c}$ is the number of allowed packet retransmissions for critical nodes.

**Figure 1:**
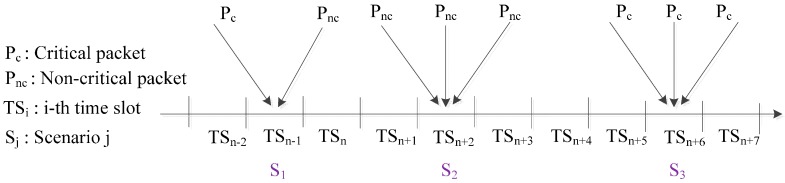
Collided packet transmission in cognitive radio (CR)-MAC.

In the CR-MAC protocol, packet collision scenarios are defined in three states, as shown in Figure 1:

- S_1_: One critical packet arrival of transmission power level P_2_ and one or more non-critical packet arrivals of P_1_;
- S_2_: More than one non-critical packet arrival;
- S_3_: More than one critical packet arrival.

In S_1_, the collision is partial due to the critical packet transmission being considered successful because of the cognitive radio transmission feature of CR-MAC. However, collisions occur between non-critical packets, as well. The states S_2_ and S_3_ involve the simultaneous arrival of more than one non-critical packet and more than one critical packet, respectively.

**Figure 2:**
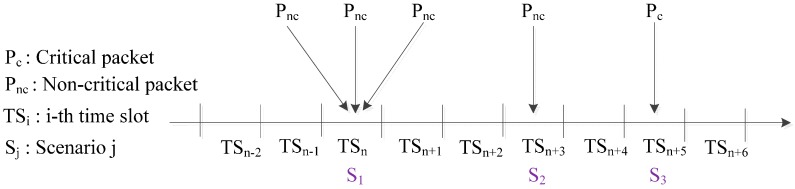
Successful packet transmission in CR-MAC.

In the CR-MAC protocol, there are three successful packet transmission scenarios, as shown in Figure 2:

- Single critical packet arrival and the arrival of one or more non-critical packets in the scenario represented by state S_1_
- Single non-critical packet arrival, in the scenario represented by state S_2_
- Single critical packet arrival, represented by state S_3_

CR-MAC achieves not only relatively high throughput, but also QoS promising for traffic of different priority classes. In CR-MAC, the traffic of sensor nodes is classified into critical traffic and non-critical traffic. After critical traffic throughput is first analyzed, the output is then applied to the analysis of non-critical traffic throughput, which depends on the transmission of critical traffic. Critical and non-critical traffic are prioritized according to transmission power. The analytical and simulation-based results disclosed different practical design approaches as follows: In critical nodes, when the number of packet retransmissions increases, the throughput increases and the number of packet rejections decreases. In non-critical nodes, the throughput decreases and the packet rejection rate increases. When the number of packet retransmissions in critical nodes reaches a certain value, the throughput of critical and non-critical nodes reaches a saturation point.

### 3.2. Dynamic Channel Adjustable Asynchronous Cognitive Radio MAC Protocol

Because of the fast channel switching capability of asynchronous MAC, the dynamic channel adjustable asynchronous cognitive radio MAC (DCAA-MAC) protocol [16] provides low latency, energy efficiency, configurability and no need for synchronization in wireless medical body area networks.

At initialization, each node scans and selects a channel with the best conditions (e.g., low signal-to-noise ratio (SNR), least frequently used, *etc.*). Each node in the network goes to sleep and wakes up periodically and independently. A node first sends a preamble; then, after receiving the acknowledgment (ACK) message from the destination node, it sends the data packets. On the receiver side, the receiver detects the preamble and remains woken up in order to receive the data. Once transmission is complete, both the sender and receiver nodes go to sleep mode. If the node detects interference (appearance of noise or PUs) on its channel, it switches to another available channel for effective communication.

Multiple channels allow the maintenance of effective communication even upon the appearance of PUs. Nodes detect signals with greater frequency than the threshold during clear channel assessment (CCA) with a fast sensing period on the current channel and listen to signals to find the address of the destination periodically. If the address of the destination matches its own address, the node sends the ACK message; otherwise, it goes to sleep mode. If the address of the destination is not decoded, it determines that there is interference or that PUs have appeared and switches to another channel for PU protection and QoS provision. The node switches to a control channel and listens to find another node that is broadcasting a channel switching preamble (CSPreamble). After receiving the preamble, the node transmits the channel switching ACK (CSACK) message to the source node and switches to the channel.

Spectrum sensing is one of the major requirements of cognitive radio. Energy detection and feature detection are the most commonly used techniques for spectrum sensing in the CR environment. In feature detection, the presence of primary users is determined by extracting specific features. The feature detection technique is most effective for CR networks, but requires significantly long sensing times and is computationally complex. Energy detection is the optimal solution to sense the presence/absence of PUs. In DCAA-MAC, channel switching is performed on the basis of energy detection. The fast channel switching mechanism provides QoS for medical body area sensor networks.

In DCAA-MAC, PU protection and QoS provision are considered, that is, a node switches to another channel if the address of the destination is not decoded, by assuming that PUs have appeared. Channel switching is performed based on energy detection to provide QoS. Each node goes to sleep mode and wakes up periodically and independently of others. DCAA-MAC has low energy consumption and low latency and allows coexistence with simultaneously operating and independent networks.

### 3.3. Asynchronous MAC Protocol for Spectrum Agility

Energy consumption plays a major role in the design of wireless body area sensor networks (WBASNs). The wireless spectrum in ISM bands becomes crowded and causes coexisting interference. High coexisting interference in networks causes high energy consumption, increased packet delay and low network throughput. An energy-efficient MAC protocol, the cognitive-receiver initiated cycled receiver (C-RICER), was designed for WBASNs in high interference environments [17]. C-RICER adjusts both channel frequency and transmission power to reduce interference and energy consumption. The main purpose of applying CR is to maintain the SNR for data exchange and to overcome the disadvantages of high coexisting interference in WBANs. By dynamically scanning and switching channels, WBANs can avoid coexisting interference, save energy and reduce packet drops and packet delays.

The energy required for the cognitive task of C-RICER is mainly consumed at the coordinator. The spectrum agility function of C-RICER is considered to be a very important characteristic of WBANs. The use of a transceiver-per-sensor node with energy constraints renders the design of C-RICER more challenging and different from traditional cognitive MAC protocols. Traditional MAC protocols are divided into two families depending on how a node gains medium access: contention-based and reservation-based protocols.

The contention-based MAC is more suitable for WSNs, because of limits on traffic and energy [18]. Receiver-initiated protocols in the contention-based family seem more suited to the context of WBANs. A well-known protocol in this family is the RICER protocol; RICER3b and RICER5b are variants of RICER according to the number of required handshakes. In the RICER3b protocol, the destination node with no data packet to transmit wakes up periodically and transmits a short wakeup beacon to indicate that it is awake. Having transmitted the beacon, it monitors the channel for a response; if there is no response, the node goes back to sleep mode. A source node with data to transmit stays awake, monitors the channel and awaits a wakeup beacon from the destination nodes.

The use of beacons in RICER3b is different than that in ZigBee [19]. In ZigBee, the beacons are used to synchronize the superframes of nodes. The data are transmitted using the carrier sense multiple access with collision avoidance (CSMA-CA) algorithm based on an appropriate back-off timer. However, in the case of RICER3b, having received a wakeup beacon, the source node instantly sends back a buzz signal and starts transmitting data. Having received the buzz signal, the destination stays awake and waits for data from the source node.

**Figure 3:**
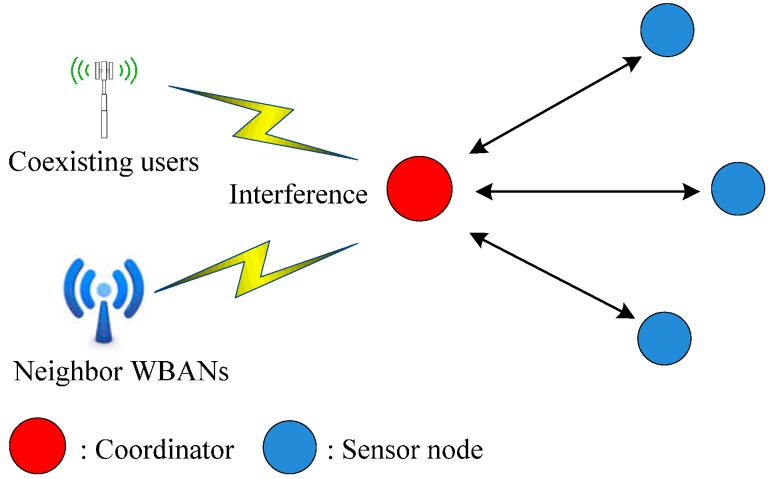
Conceptual operation in cognitive-receiver initiated cycled receiver (C-RICER).

The main objective of C-RICER is early detection of interference in its working channel. Channel sensing is periodically performed at the coordinator. According to the detected level of interference, the WBASN can adaptively switch to the channel with the lowest interference to prevent coexisting interference. The conceptual operation in C-RICER is shown in Figure 3. In C-RICER, sensing and switching is continuously performed during the operation. Channel inferences can be unpredictable, which prompts frequent channel switching and costs a considerable amount of energy. To address this, C-RICER uses power adaption methods prior to considering channel adaption. The working mechanism of C-RICER involves two tasks: a data exchange task and a cognitive task. The data exchange task is used for data collection, and the cognitive task is used to dynamically adapt both the transmission power and the channel according to the interference levels in question.

Data communication in C-RICER is based on RICER3b, with the destination node as the coordinator and the source as the sensor node. The coordinator wakes up and sends a wakeup beacon. The sensor node receives the beacon and transmits a buzz signal to caution the coordinator to wait for data. Having received the buzz signal, the coordinator waits for data. The sensor node sends the data and waits for the ACK signal. The coordinator receives the data and sends the ACK to the sensor node. The cognitive task of C-RICER has three main features: channel sensing, power adaption and channel adaption.

Channel sensing is the initial step in detecting interference. The received signal strength indicator (RSSI) is used to measure the interference level in different channels. In WBASNs, the coordinator is the node in the network most suited to channel sensing. It is the only node that has less energy constraints than sensor nodes in the network; thus, exploiting the energy at the coordinator instead of sensor nodes can prolong the network’s lifetime. Communication is only established after a transmitter has successfully received a wakeup beacon from a receiver. The coordinator of C-RICER periodically senses the interference level only at its current channel instead of in the entire frequency band in order to reduce the required sensing energy. The coordinator scans for the interference level of the remaining channels only when the interference level of its current channel is greater than a threshold value.

**Figure 4:**
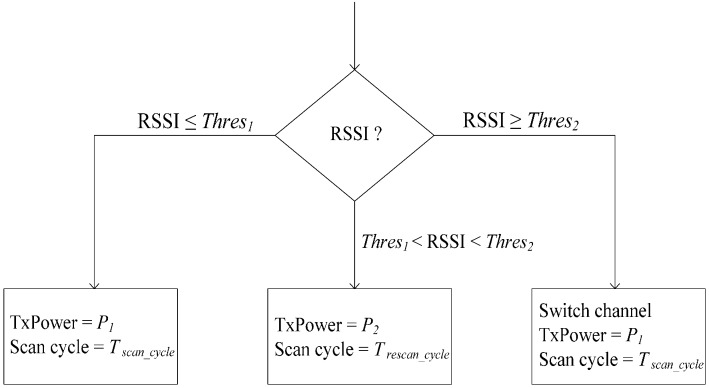
Power adaption strategy in C-RICER. RSSI, received signal strength indicator; TxPower, transmission power.

Not only can increasing the transmission power enhance SNR, it can also reduce retransmissions. As shown in Figure 4, transmission powers (TxPowers) *P_1_* and *P_2_* (*P_1_* < *P_2_*) and interference threshold values *Thres_1_* and *Thres_2_* are specified in the transceiver at each node. The transceiver of each sensor node will work at its default transmission power *P_1_* if the RSSI of the current working channel is less than *Thres_1_*. When the RSSI of the current channel is greater than *Thres_1_*, but less than *Thres_2_*, TxPower increases to *P_2_*. Once *P_2_* is used, the coordinator will adaptively change its scanning cycle by *T_rescan___cycle_* seconds to rescan the channel.

If the RSSI of the current channel is less than *Thres_1_*, the network will return to using *P_1_*. The coordinator scans the remaining channels to build the interference map when the RSSI is higher than *Thres_1_*. If the RSSI of the current working channel is greater than *Thres_2_*, the coordinator also scans the remaining channels to build the interference map. The WBASN will switch to the channel with the lowest interference level without changing transmission power. The coordinator utilizes the wakeup beacon message to broadcast TxPower information. The sensor nodes extract the bit from the beacon message. If the bit is zero, *P_1_* is used; otherwise, *P_2_* is used.

**Figure 5:**
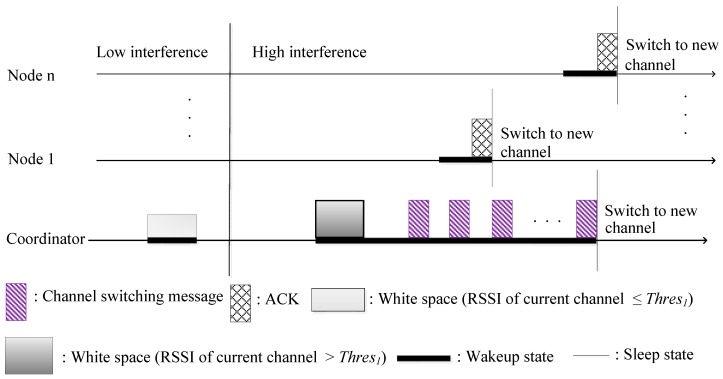
Channel switching in C-RICER.

When the interference level of the current working channel is greater than *Thres_2_*, the WBASN decides to switch to a new channel. The C-RICER coordinator sends the channel switching information to the sensor nodes. Two transceivers are used at each node: one for data transfer and the other for channel information [18]. The wakeup function of the sensor nodes depends on the demand to send data. Sensor nodes with different sensing functions will wake up randomly and will not receive the switching information simultaneously from a coordinator. A checklist table is used by the coordinator to gather information regarding sensor nodes that successfully received the switching information. Figure 5 shows the channel switching algorithm. Once a channel switching decision is made, the index of the channel with the lowest RSSI is attached to the channel switching message. The coordinator periodically broadcasts the channel switching message and waits for the ACK message from the sensor nodes. The channel switching checklist table of the coordinator is filled with successfully received ACK messages from a sensor node. If there is a data packet to transmit, the sensor nodes will wake up and wait for the wakeup beacon form the coordinator.

In C-RICER, power adaption methods are used prior to consider channel adaption because channel inferences can be unpredictable and frequent channel switching costs a considerable amount of energy. Furthermore, sensing energy is additionally reduced by sensing the interference level only at the current channel instead of in the entire frequency band. In addition, the C-RICER protocol with cognitive functions eludes interference and enhances network performance. C-RICER adjusts both channel frequency and transmission power to reduce interference and energy consumption. By dynamically scanning and switching channels, the WBANs can avoid coexisting interference, save energy and reduce packet drop and packet delay. In a high-interference environment involving high energy consumption, the C-RICER protocol can outperform RICER3b.

### 3.4. Cognitive Radio for Medical Body Area Networks Using Ultra-Wideband

Medical body area networks (MBANs) have been introduced to unlicensed frequency bands, where mutual interference between electronic devices is high. CR technology can potentially alleviate such problems in MBANs and increase the efficiency of spectrum management. In [20], CR features based on ultra-wideband (UWB) technology for MBANs were proposed by considering both the PHY and MAC layers. A WBAN comprises multiple sensor nodes capable of sampling, processing and communicating biomedical information [21]. Cognitive capabilities are implemented in the body network controller (BNC) using impulse radio (IR) and multiband orthogonal frequency-division multiplexing (MB-OFDM). Impulse radio ultra-wideband (IR-UWB) technology was introduced as a solution in IEEE 802.15.6 and is currently being developed by the IEEE 802.15.6 Task Group 6 [22].

The advantages of IR-UWB technologies are their low power, low complexity and low cost with highly reliable wireless communication. The main objective of the standard is to specify a MAC sublayer that can support several PHY layers, including UWB. IR-UWB provides high QoS, and frequency modulation UWB (FM-UWB) is optimized for low power consumption and reliable communication.

**Figure 6:**
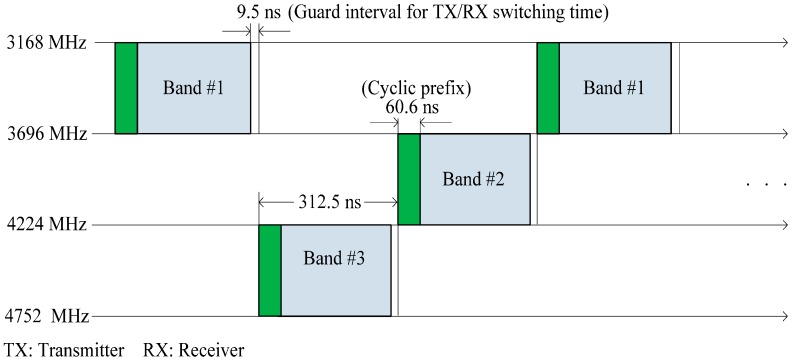
Multiband orthogonal frequency-division multiplexing (MB-OFDM).

The European Computer Manufacturers Association’s ECMA-368 specification is a high-rate UWB PHY and MAC wireless standard, uses MB-OFDM and divides the spectrum into 14 bands of 528 MHz each [23]. OFDM is based on a frequency diversity transmission scheme that distributes modulated data across closely and mutually overlapping space. An OFDM signal is generated in the frequency domain by using inverse fast Fourier transform (IFFT) to create a time domain multiplexed signal. UWB signals have an inherent noise-like behavior that makes UWB difficult to detect and requires a complex encryption algorithm. In MB-OFDM, symbols are interleaved over multiple sub-bands across time and frequency, as shown in Figure 6. By interleaving the OFDM symbols across sub-bands, multiband UWB can maintain the power level associated with a single-band OFDM. The characteristics of UWB technology can be exploited to turn a BNC into a cognitive radio controller (CRC) that controls the transmission parameter of CR clients. The communication links between the sensors and the BNC should be implemented using IR-UWB, which is specified by IEEE 802.15.6.

The architecture for CRC consists of two transceivers: an IR-UWB transceiver with on-off keying (OOK) and an MB-OFDM UWB transceiver. The division of the 3.1–10.6 GHz UWB spectrum into 14 sub-bands of 528 MHz each is adopted. The lower UWB frequency band (3.1–4.8 GHz) is enclosed with three sub-band frequencies at 3232, 3960 and 4488 MHz. The three sub-band frequencies are preferred for first-tier communication, because of their better propagation characteristics. The MB-OFDM transceiver also protects medical devices and sensors from UWB interference by using frequency domain spectrum shaping.

UWB facilitates a high processing gain and allows coexistence with a narrow wide-band system through spectrum shaping. Spectrum shaping can be achieved by adopting time hopping codes using a pulse-position modulation scheme or the pulse shape at a transmitter. The MB-OFDM UWB system provides clear advantages over IR-UWB in terms of sensing using power spectral density (PSD). A sub-band or channel ranking scheme can be implemented using a combination of two methods: using the sub-band’s PSD information to identify available channels and using bit error rate (BER) information obtained from active channels to yield channel quality [24].

To avoid interferences and collisions between signals, wireless devices need to coordinate access time periods with their neighbors. This type of coordination is governed by the MAC sublayer. The IEEE 802.15.6 standard is used for the intra-WBAN tier, whereas inter-tier access is provided by ECMA-368 [23]. IEEE 802.15.6 supports the beacon and non-beacon modes of operations. It is similar to MAC defined for IEEE 802.15.4, except for the definition of multiple phases in the time division multiple access (TDMA) frame. Here, the standard defines exclusive access phases (EAPs) for high-priority devices, such as emergency signals related to a patient’s vital signs, random access phases (RAPs) based on slotted Aloha, contention access phases (CAPs) and Type I/II phases based on polling. The ECMA-368 MAC provides a distributed reservation-based channel access mechanism, as well as a prioritized contention-based channel access mechanism. The architecture of the ECMA-368 MAC is fully distributed.

All devices provide all required MAC functions and optional functions as determined by the application. No device acts as a central coordinator. Channel time is divided into superframes, with each superframe composed of two major parts: the beacon period (BP) and the data period. The BP is organized into slots and is used to achieve network synchronization, exchange reservation and scheduling information for medium access purposes. During the data period, devices send and receive data using prioritized contention access (PCA) or in reservations established using the distributed reservation protocol (DRP).

Although beacons make network operations significantly simpler, it should be noted that beacon-less operation is possible, as well, by relying on header extensions within the physical layer service data unit used for piggybacking control information. The CRC sends the following information to the IR-UWB devices: schedule of sensing and data reporting and input of selected pulse shapes according to the results of sensing. The 802.15.6 MAC operates using TDMA and supports beacon mode, where the BNC sends broadcast information towards the WBAN devices followed by a TDMA frame. The beacon can be modified to include shaping information (e.g., pulse shape, code parameter, *etc.*) for IR-UWB devices.

This protocol functionally adopts IEEE 802.15.6 for communication in intra-WBAN tiers and ECMA-368 in inter-WBAN tiers. The distributed reservation-based channel access mechanism is also supported in addition to the prioritized contention-based channel access mechanism. Of course, the design of the MAC protocol for a CR network is among the challenging tasks if the network needs to operate in a distributed fashion [25]. Further research is needed to manage the multiple radio access technologies (RATs) available in CRC.

### 3.5. HCVP: Hybrid Cognitive Validation Platform for WBANs

Because of their complexity and energy inefficiency, field-programmable gate arrays (FPGAs) are not reliable for wireless networks, like WBANs. Instead, a hybrid cognitive validation platform (HCVP) [26] was proposed by integrating software and hardware devices. An adaptive CR-MAC algorithm was implemented to minimize the impact of interference. HCVP is based on programmable system-on-chip (SoC) processors and provides a simulation environment close to the practical scenario.

**Figure 7:**
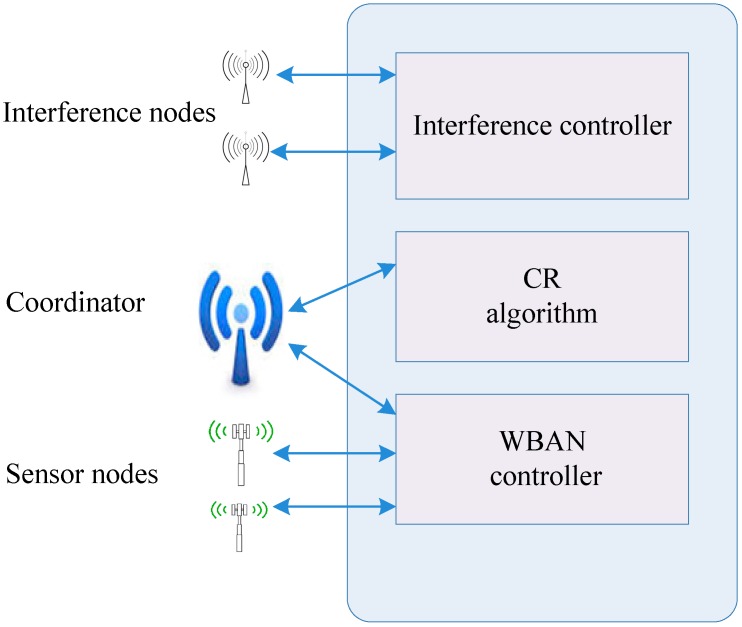
System architecture of hybrid cognitive validation platform (HCVP).

Network simulation tools, such as network simulator version 2 (NS-2), optimized network engineering tool (OPNET), *etc.*, are widely used and are convenient to develop, deploy, debug and modify different algorithms and parameters. However, the simulation software faces several challenges, such as modifying the PHY layer, which is very difficult to accurately implement. The HCVP is composed of two parts, the lower part and the upper part. The lower part consists of the PHY and MAC layers, and the upper part comprises the CR algorithm and the network layer. Figure 7 shows the system architecture of HCVP. The interference controller generates traffic distribution for the evaluation. In HCVP, the workflow of a CRBAN includes the WBAN control process and the CR process.

**Figure 8:**
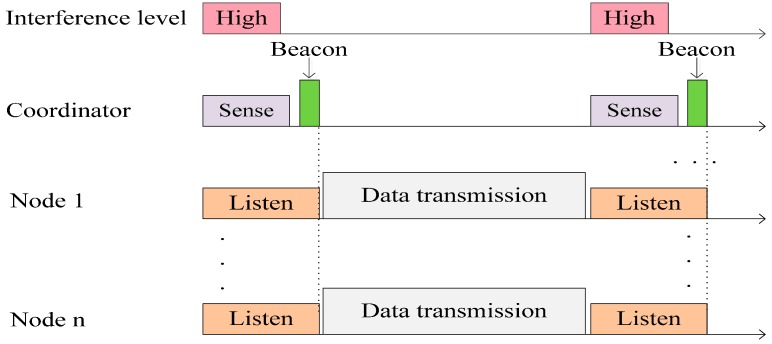
Adaptive CR-MAC diagram.

To mitigate coexisting interference, an adaptive CR MAC is implemented, as shown in Figure 8. The coordinator node is equipped with a high-capacity battery. The nodes in a CRBAN form a star topology with the central coordinator node. The coordinator node assumes the responsibility of managing the entire CRBAN, as well as CR functions. The CR algorithm analyzes the channel parameters according to sensing results, estimates the idle time and passes the result on to the network controller. The network coordinator broadcasts a beacon with the information regarding each superframe. Having received a beacon, member nodes attempt to access the channel during a superframe.

For the implementation of HCVP, a programmable wireless SoC CC2510 was used. CC2510 is a high-performance, low-cost, low power consumption 2.4-GHz system SoC designed for power-critical wireless systems. This chip is integrated with a microcontroller, memory, RF transceiver and other common interferences, such as the universal synchronous/asynchronous receiver/transmitter (USART). In this system, the MAC layer is bound to the firmware of SoC, whereas the PHY layer corresponds to the RF module. To evaluate the performance of the adaptive MAC algorithm, two different traffic models are used. The exponential distribution model is applied to generate same-frequency interference. Four categorized priority levels of the traffic model are established based on real applications, such as mobile healthcare. Without CR functions, the collision rate increases linearly according to the increase in interference traffic.

The HCVP platform effectively integrates the hardware and software with the PHY and MAC layers working on practical hardware devices and the CR algorithm. That is, HCVP is based on programmable SoC processors, in which a simulation environment close to the practical scenario is provided, as well. The network throughput is greatly improved by implementing the CR algorithm, because the collision rate gradually decreases when the CR algorithm is introduced. The channel estimation is effectively carried out on the basis of the RSSI measured during the sensing period and the packet collision rate during the WBAN accessing period. HCVP achieves excellent performance on energy efficiency and is suitable for the validation of power-critical applications.

## 4. Comparison of MAC Protocols

In this section, we compare and discuss the MAC protocols designed for CRBANs. In Table 1, the MAC protocols reviewed in Section 3 are compared with respect to the collision ratio, channel access parameters, energy consumption, advantages and limitations.

**Table 1:** Comparison of MAC protocols designed for CRBANs. DCAA, dynamic channel adjustable asynchronous; PU, primary user; MBAN, medical body area network; UWB, ultra-wideband; ECMA, European Computer Manufacturers Association.

Protocol	Collision ratio	Channel access parameter	Energy consumption	Advantages	Limitations
CR-MAC [15]	Low	Energy level detection	Low	High throughput. According to the urgency level of the monitored traffic, nodes are assumed to have the capability to dynamically tune their transmitter power. QoS promising for traffic of different priority classes.	No advantage in increasing the number of retransmissions in critical nodes after saturation point.
DCAA-MAC [16]	Low	Energy level detection	Low	PU protection and QoS provision. Each node goes to sleep and wakes up periodically and independently.	Energy detection is only optimal for detecting unknown signal if the noise power is known.
C-RICER [17]	Low	RSSI	Low	Use of power adaption before channel adaption is considered. Dynamically adapts both transmission power and channel according to interference level. To reduce the sensing energy, the coordinator of C-RICER periodically senses interference only in the working channel.	Random delay. Rescan cycle should be sufficient to cover the duration of the interference, but sufficiently short to ensure energy efficiency. Thus, the rescan cycle should be adaptively calculated.
MBAN MAC [20]	Low	UWB radio	Low due to IR-UWB	Adopts the IEEE 802.15.6 for communication in intra-WBAN tiers and ECMA-368 in inter-WBAN tiers. ECMA-368 MAC provides a distributed reservation-based channel access mechanism, as well as prioritized contention-based channel access.	Neither verified nor evaluated in [20].
HCVP MAC [26]	Low	RSSI	Medium	Realizes practical situations by integrating computer software and hardware devices. Channel estimation is based on both the RSSI measured during the sensing period and the packet collision rate during the WBAN accessing period.	The coordinator broadcasts beacons only when suitable time slots are found (*i.e*., no interference); hence, general nodes keep waiting for beacons.

In [15], packet transmission for critical nodes was prioritized over that for non-critical nodes by using the concept of cognitive radio. Critical packets were transmitted with higher power, whereas non-critical packets were transmitted with lower power. As a result, the throughput of critical traffic was higher than that of non-critical traffic. The packet collision ratio was low because of the preference accorded to critical traffic over non-critical traffic. According to the performance results of network throughput and the packet rejection rate, CR-MAC [15] provided QoS support for traffic with different priorities.

In DCAA-MAC [16], the channel access mechanism is performed on the basis of energy detection. To provide energy efficiency, each node goes to sleep and wakes up periodically and independently. The main advantages of this protocol are low latency, energy efficiency, configurability and no synchronization. Furthermore, the fast channel switching mechanisms provide QoS for CRBANs through low latency. Energy detection is useful in detecting the signal when the noise level is known. By implementing feature detection in CRBANs, the presence of PU signals can be effectively determined by extracting specific features of traffic.

C-RICER [17] works cognitively and energy efficiently in high-interference environments. The interference level of a wireless channel is measured by using RSSI values. To prevent coexisting interference, the affected working channel is adaptively switched to the lowest-interference channel. The data collision ratio is low because of the continuous sensing and switching of channels. The main feature of C-RICER is that it senses interference only in the current channel rather than the entire frequency band in order to reduce energy consumption. To render the system more reliable and energy efficient, the rescan cycle time should be adapted according to the interference duration.

In MBAN MAC [20], the cognitive capabilities are implemented in the network controller. The main features of the protocol are low power, low complexity and low cost with highly reliable communication. The protocol is energy efficient, because of the use of IR-UWB. In [20], several cognitive radio techniques were applied to UWB MBANs to improve coexistence with other systems through frequency agility and frequency domain spectrum shaping.

HCVP [26] provides an evaluation environment that provides a closer approximation to practical situations than other network simulation tools. Channel estimation is based on RSSI values, which are measured during the sensing period. The packet collision rate is reduced and the throughput is greatly improved when the CR algorithm is introduced. HCVP is a good solution to realize practical network scenarios by integrating software and hardware. Moreover, it is easier to deploy and configure than FPGA-based platforms. By choosing low-power SoC chips, HCVP achieves energy-efficient performance and is suitable for testing power-critical applications.

Among the five MAC protocols designed for CRBANs, MBAN MAC [20] may be the primary choice when the target application area is remote healthcare monitoring. For example, patients are continuously monitored by sensors embedded on their bodies, and the sensed data are collected by a WBAN coordinator. The data collected by the WBAN coordinators are transferred to a remote monitoring system. The protocol resolves different issues and efficiently exploits cognitive radio technology. Furthermore, the protocol can adopt IEEE 802.15.6 for communication in intra-WBAN and ECMA-368 for inter-WBAN tiers. Further, the ECMA-368 MAC provides a distributed reservation-based channel access mechanism, as well as prioritized contention-based channel access.

## 5. Open Research Issues and Challenges

In this section, we present issues and challenges encountered in designing reliable systems and state open research problems in the systems surveyed in Section 3. There are numerous challenges in implementing a cognitive radio environment in a wireless body area network, a few of which are as follows:

### 5.1. Implementing Cognitive Radio Attributes to Sensors

Radio-equipped sensors with cognitive capabilities may incur higher energy consumption, while performing cognitive actions, such as learning, sensing and adapting. Secondary users have to switch their respective channel frequencies and transmission powers according to the type of operating network (e.g., IEEE 802.11 [27] or IEEE 802.16 [28]). For a cognitive environment, all network devices related to sensors and other controllers may efficiently perform cognitive operations, but increase the design complexity of network devices. A number of CRBAN architectures have been proposed, as detailed in Section 3, where cognitive attributes have been applied to the network controller rather than the sensors. CRBANs will become fully cognitive by introducing cognitive attributes to all sensors, which will help sensors make independent decisions in order to reduce interference and improve QoS [29].

### 5.2. Power Consumption and Energy Harvesting

In CRBANs, the replacement of batteries is not practical. CR sensors are power-constrained devices. In addition to the energy needed for normal network operations (such as route discovery, transmission/reception of data packets and data processing), CR sensors also require power for spectrum sensing, channel negotiation and frequent spectrum handoffs. Energy harvesting will have a significant impact on the lifetime of future CRBANs. Common sources of energy harvesting include mechanical, thermal, electromagnetic, natural energy and energy from the human body. Energy harvesting devices available nowadays can efficiently capture, accumulate and store energy to power sensor nodes [30]. The source of energy can be utilized by means of an energy-efficient protocol to extend network life.

### 5.3. Reducing Interference

Accurate detection of the physical locations of biomedical devices can help reduce mutual interference. A radio-frequency identification (RFID)-based transceiver was introduced in [6] to reduce interference in biomedical devices by detecting their physical location. RFID transceivers may be suitable for CRBANs because of their low power transmission, but they can cause interference in biomedical devices [31]. Without the accurate physical location offered by RFID, reducing mutual interference between primary and secondary users becomes a significant challenge. CRBANs can be designed using physical location information or other approaches to mitigate interference.

### 5.4. Security and Privacy

Security and privacy issues are major concerns in every field. CRBANs enable the collection of critical data, and medical personnel use these to provide better services. However, the devices used present unique security and privacy challenges. Highly secured authentication and encryption mechanisms must be applied to prevent eavesdropping and other intrusions. Addressing security in these systems involves numerous difficulties. In CR networks, a node must carry out cognitive actions (such as learning, sensing and adapting) in its operating environment, which can be manipulated by attackers. CRBANs inherit most of the well-known security challenges from wireless sensor networks (WSNs). However, the typical characteristics of CRBANs, such as severe resource constraints and harsh environmental conditions, pose additional challenges for security and privacy support [32].

### 5.5. Networking with Cognitive Radio Capability

In the CR paradigm, unlicensed secondary users can access some licensed parts of radio spectra. Some CR schemes have been studied for medical wireless body area networks [5,33]. Energy efficiency, collision avoidance and interference cancellation are the main goals in CRBANs for ubiquitous health monitoring. A considerable amount of current research is focusing on the MAC layer. Network layer and cross-layer design are other promising research areas that need to be addressed more extensively in the future.

### 5.6. Enhancing QoS

QoS provision is also a major concern. QoS requirements vary according to application and operating environment. QoS support is a challenging issue because of the numerous resource constraints, such as limited power, bandwidth, memory, processing power, *etc.*, in CRBANs. Another challenge in CRBANs is to protect the rights of PUs to access incumbent spectra. Primary users’ communication should be free of interference from secondary users. Priority-based scheduling schemes can be applied to provide context-based QoS differentiation. A greater challenge is accurately predicting a PU’s arrival at the channel. False alarms and missed detections of primary users pose further challenges in CRBANs.

## 6. Conclusions

In this paper, we have detailed, reviewed and compared existing MAC protocols for CRBANs. High spectrum utilization, reliable communication between nodes, minimum delay and low energy consumption are key parameters for efficient MAC protocols. One MAC protocol cannot satisfy the requirements of all applications, because the protocols in CRBANs are hardware- and application-dependent. CR technology in CRBANs can effectively reduce EMI through efficient spectrum management and enhance QoS by considering the priority level of medical sensors. The main advantage of CRBANs as compared to WBANs is their flexibility. In CRBANs, it is possible to support delay-sensitive traffic, and the traffic can also be prioritized to improve delivery latency. The urgent message should always have the highest priority, and the normal transmission can use the necessary resources after serving the QoS-guaranteed traffic. The traffic priorities may change from time to time, depending on the types of data. The energy-efficient dynamic resource allocation, transmission scheduling and security are the key issues in CRBANs, as well. Our work here will be useful for researchers attempting to develop novel and low-power MAC protocols for CRBANs.

## References

[1] Movassaghi S., Abolhasan M., Lipman J., Smith D., Jamalipour A. (2014). Wireless Body Area Network: A Survey. IEEE Commun. Surveys Tuts..

[2] Kwak K.S., Ameen M.A., Kwak D., Lee C., Lee H. A Study on Proposed IEEE 802.15 WBAN MAC Protocols. Proceedings of the 9th International Symposium on Communications and Information Technology (ISCIT’09).

[3] Salman N., Rasool I., Kemp A.H. Overview of the IEEE 802.15.4 standards family for low rate wireless personal area networks. Proceedings of the 7th International Symposium on Wireless Communication Systems(ISWCS).

[4] Haykin S. (2005). Cognitive Radio: Brain-Empowered Wireless Communications. IEEE J. Select. Areas Commun..

[5] Shan F., Zhongliang L., Dongmei Z. (2010). Providing Telemedicine Services In An Infrastructure-Based Cognitive Radio Network. IEEE Wirel. Commun..

[6] Phunchongharn P., Hossain E., Niyato D., Camorlinga S. (2010). A Cognitive Radio System for e-health Applications in a Hospital Environment. IEEE Wirel. Commun..

[7] Cavallari R., Martelli F., Rosini R., Buratti C., Verdone R. (2014). A Survey on Wireless Body Area Networks: Technologies and Design Challenges. IEEE Commun. Surv. Tuts..

[8] Kartsakli E., Lalos A.S., Antonopoulos A., Tennina S., Renzo M.D., Alonso L., Verikoukis C. (2014). A Survey on M2M Systems for mHealth: A Wireless Communications Perspective. Sensors.

[9] Rahim A., Javaid N., Aslam M., Rahman Z., Qasim U., Khan Z.A. A Comprehensive Survey of MAC Protocols for Wireless Body Area Networks. Proceedings of the 7th International Conference on Broadband, Wireless Computing, Communication and Applications (BWCCA).

[10] Xiang J., Zhang Y., Skeie T. (2010). Medium access control protocols in cognitive radio networks. Wirel. Commun. Mob. Com..

[11] Ye W., Heidemann J., Estrin D. An Energy-Efficient MAC Protocol for Wireless Sensor Networks. Proceedings of the 21st Annual Joint Conference of the IEEE computer and Communications Societies.

[12] El-Hoiydi A., Decotignie J.-D. WiseMAC: An Ultra-Low Power MAC Protocol for the Downlink of Infrastructure Wireless Sensor Networks. Proceedings of the 9th IEEE Symposium on Computers and Communication (ISCC’04).

[13] Nan H., Hyon T.-I., Yoo S.-J. Distributed Coordinated Spectrum Sharing MAC Protocol for Cognitive Radio. Proceedings of the 2nd IEEE International Symposium on New Frontiers in Dynamic Spectrum Access Networks, (DySPAN 2007).

[14] Niyato D., Hossain E. (2009). Cognitive radio for next-generation wireless networks: An approach to opportunistic channel selection in IEEE 802.11-based wireless mesh. IEEE Wirel. Commun..

[15] Ali K.A., Sarker J.H., Mouftah H.T. A MAC protocol for cognitive wireless body area sensor networking. Proceedings of 6th International Wireless Communications and Mobile Computing Conference.

[16] Lee B., Yun J., Han K., Kim T.-H., Adeli H., Fang W.-C., Vasilakos T., Stoica A., Patrikakis C., Zhao G., Villalba J., Xiao Y. (2011). Dynamic Channel Adjustable Asynchronous Cognitive Radio MAC Protocol for Wireless Medical Body Area Sensor Networks. Communication and Networking.

[17] Nhan N.-Q., Gautier M., Berder O. Asynchronous MAC protocol for spectrum agility in Wireless Body Area Sensor Networks. Proceedings of the 9th International conference on Cognitive Radio Oriented Wireless Networks and Communications (CROWNCOM).

[18] De Domenico A., Strinati E.C., di Benedetto M. (2012). A survey on MAC strategies for Cognitive radio networks. IEEE Commun. Surv. Tuts..

[19] Ramya C., Shanmugaraj M., Prabakaran R. Study on ZigBee technology. Proceedings of the 3rd International Conference on Electronics Computer Technology (ICECT).

[20] Chávez-Santiago R., Nolan K.E., Holland O., de Nardis L., Ferro J.M., Barroca N., Borges L.M., Velez F.J., Gonçalves V., Balasingham I. (2012). Cognitive Radio for Medical Body Area Networks Using Ultra Wideband. IEEE Wirel. Commun..

[21] Chen M., Gonzalez S., Vasilakos A., Cao H., Leung V.C. (2011). Body Area Networks: A Survey. Mob. Netw. Appl..

[22] Lee C., Kim J., Lee H.S., Kim J. Physical Layer Designs for WBAN Systems in IEEE 802.15.6 Proposals. Proceedings of the 9th International Symposium on Communications and Information Technology (ISCIT 2009).

[23] ECMA-368 Standard, High Rate Ultra Wideband PHY and MAC Standard 2005. http://www.ecma-international.org/publications/files/ECMA-ST/ECMA-368.pdf.

[24] Hossian M., Mahmood A., Jantti R. Channel Ranking Algorithms for Cognitive Coexistence of IEEE 802.15.4. Proceedings of the IEEE 20th International Symposium on Personal, Indoor and Mobile Radio Communications.

[25] Jha S.C., Rashid M.M., Bhargava V.K., Despins C. (2011). Medium Access Control in Distributed Cognitive Radio Networks. IEEE Wirel. Commun..

[26] Han J., Liu J., Yu H., Chen C., Shen Z. HCVP: A Hybrid Cognitive Validation Platform for WBAN. Proceedings of the International Conference on Wireless Communications & Signal Processing (WCSP).

[27] Crow B.P., Widjaja I., Kim J.G., Sakai P.T. (1997). IEEE 802.11 wireless local area networks. IEEE Commun. Mag..

[28] Hossain E. IEEE802.16/WiMAX-Based Broadband Wireless Networks: Protocol Engineering, Applicatio-ns, and Services. Proceedings of the 5th Annual Conference on Communication Networks and Services Research (CNSR ’07).

[29] Yu R., Zhang Y., Gao C., Huang C., Gao R. (2011). Energy-efficient and Reliability-driven Cooperative Communication in Cognitive Body Area Networks. Mob. Netw. Appl..

[30] Barroca N., Ferro J., Borges L., Tavares J., Velez F. Electromagnetic Energy Harvesting for Wireless Body Area Networks with Cognitive Radio Capabilities. Proceedings of the URSI Seminar of the Portuguese Committee.

[31] Van Der Togt R., van Lieshout E.J., Hensbroek R., Beinat E., Binnekade J., Bakker P. (2008). Electromagnetic Interference from Radio Frequency Identification Inducing Potentially Hazardous Incidents in Critical Care Medical Equipment. J. Am. Med. Assoc. (JAMA).

[32] Li M., Lou W.J., Ren K. (2012). Data Security and Privacy in Wireless Body Area Networks. IEEE Wirel. Commun..

[33] Syed A.R., Yau K.-L.A. On Cognitive Radio-Based Wireless Body Area Networks for Medical Applications. Proceedings of the IEEE Symposium on Computational Intelligent in Healthcare and e-health (CICARE 2013).

